# Detection of Rail Defects Using NDT Methods

**DOI:** 10.3390/s23104627

**Published:** 2023-05-10

**Authors:** Longhui Xiong, Guoqing Jing, Jingru Wang, Xiubo Liu, Yuhua Zhang

**Affiliations:** 1 Postgraduate Department, China Academy of Railway Sciences, Beijing 100081, China; 2 Infrastructure Inspection Research Institute, China Academy of Railway Sciences Co., Ltd., Beijing 100081, China; 3 School of Civil Engineering, Beijing Jiaotong University, Beijing 100044, China

**Keywords:** rail defect, comprehensive detection, NDT, integrated system, in-service, high-speed

## Abstract

The rapid development of high-speed and heavy-haul railways caused rapid rail defects and sudden failure. This requires more advanced rail inspection, i.e., real-time accurate identification and evaluation for rail defects. However, existing applications cannot meet future demand. In this paper, different types of rail defects are introduced. Afterwards, methods that have the potential to achieve rapid accurate detection and evaluation of rail defects are summarized, including ultrasonic testing, electromagnetic testing, visual testing, and some integrated methods in the field. Finally, advice on rail inspection is given, such as synchronously utilizing the ultrasonic testing, magnetic flux leakage, and visual testing for multi-part detection. Specifically, synchronously using the magnetic flux leakage and visual testing technologies can detect and evaluate surface and subsurface defects, and UT is used to detect internal defects in the rail. This will obtain full rail information, to prevent sudden failure, then ensure train ride safety.

## 1. Introduction

Railway track, used to support vehicle operation, is a crucial component of the whole railway system (including, e.g., vehicle, catenary, subgrade, bridge, etc.). The track component, rail, plays an indispensable role in safe train operation and ride comfort [1]. Therefore, it is vital to have timely rail inspection through various devices based on non-destructive testing (NDT) methods [2].

Traditional manual inspection methods are time-consuming, laborious, and, most importantly, affected by various factors, such as inspector experience and complex environmental conditions, while NDT rapid detection for rail defects is able to perform standardized and high-efficiency inspection which could less interrupt the regular operation [3,4,5].

Normal NDT rail defect detection consists of three steps: search, identification, and evaluation. The search step requires cooperating with various NDT means to cover all parts of the rail (e.g., rail surface, sub-surface, rail head, internal part, rail foot, etc.). Identification is calculating the results based on quantitative characteristics of the NDT data. Finally, evaluation is estimating the rail defects and classifying their severity according to criteria and indicators.

There will be more intense demands for rapid and accurate detection of rail defects comprehensively. Before achieving the aim, it is essential to evaluate methods against existing field applications to provide scientifical and valid guidance.

In this paper, the rapid rail defect detection technologies are summarized. Based on that, the advantages and disadvantages of various NDT methods can be found. Afterwards, by integrating the advantages of various NDT methods, a comprehensive detection guideline is discussed.

The structure of this paper is as follows. Section 1 introduces common rail defects and popular NDT methods. Section 2, Section 3 and Section 4, respectively, discuss ultrasonic testing (UT), electromagnetic testing (ET), and visual testing (VT), including their principles and related research on field applications. Section 5 presents an integrated method for high-speed detection. Finally, Section 6 gives conclusions and perspectives for NDT rail inspection.

### 1.1. Rail Defects

Rail defects can be classified as internal defects, surface defects, or subsurface defects based on their location of occurrence. While every country follows its own classification standard, the common types of defects are generally the same.

**Figure 1:**
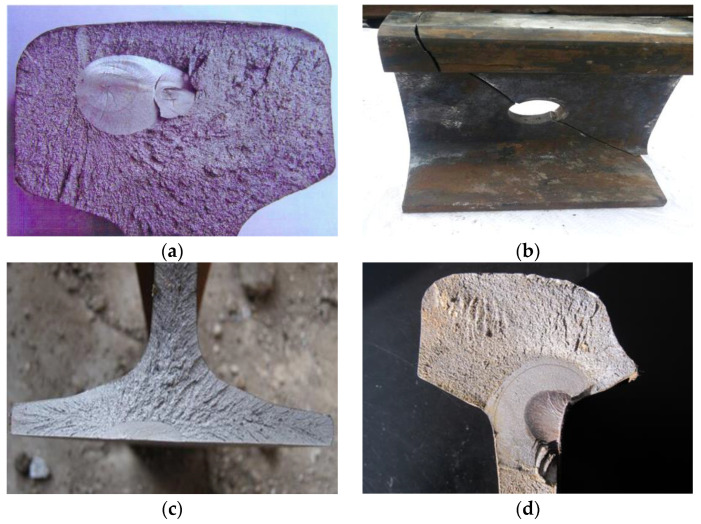
Rail internal defects: (**a**) transverse defect; (**b**) bolt hole crack; (**c**) rail bottom crack; (**d**) rail fillet transverse cracks.

Internal defects arise from inside the rail, including transverse defects, bolt hole cracks, rail bottom cracks, rail fillet transverse cracks, and others (as shown in Figure 1). These defects develop secretly and can cause dangerous derailments.

**Figure 2:**
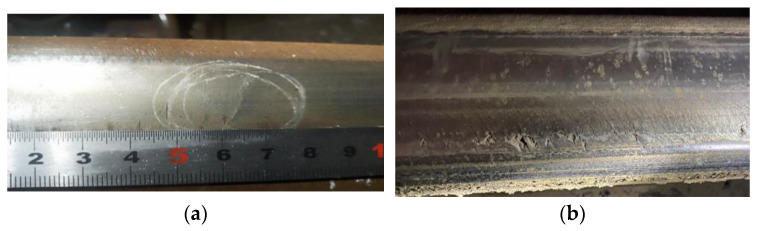

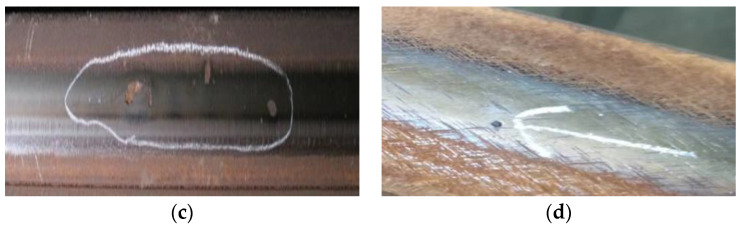
Rail surface defects: (**a**) squat; (**b**) head checking (HC); (**c**) burn; (**d**) rail indentation.

Surface and subsurface defects, on the other hand, occur on or just below the surface of the rail and include squats, head checking (HC), burns, rail indentations, and others (as shown in Figure 2). Most HC defects are classified as rolling contact fatigue (RCF) crack, which is one of the most threatening type of surface defects. Surface defects can interfere with ultrasonic rail flaw detection, making it possible for the rail to fail to detect internal defects.

### 1.2. NDT for Rail Defect Detection

**Figure 3:**
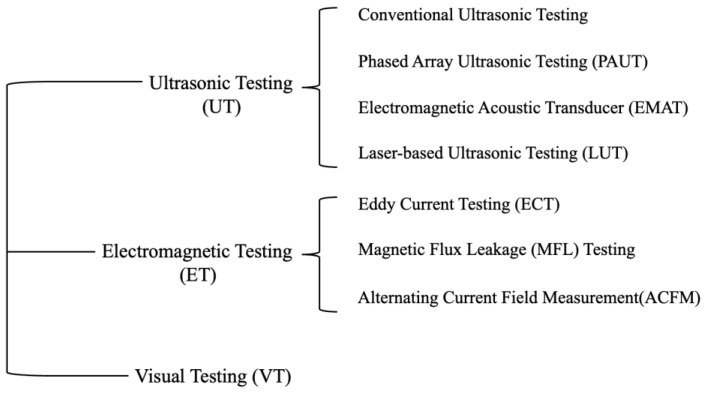
NDT for rail defect detection.

NDT, also known as non-destructive examination, non-destructive inspection (NDI), and non-destructive evaluation (NDE), is a type of inspecting or testing that is used to evaluate the properties, e.g., discontinuities of a material, component, or system without causing damage [3]. In high-speed rail defect detection, the most commonly used NDT methods are divided into three categories: ultrasonic testing (UT), electromagnetic testing (ET), and visual testing (VT) [4,6,7], as shown in Figure 3.

UT methods include conventional ultrasonic testing, phased array ultrasonic testing (PAUT), electromagnetic acoustic transducer (EMAT), and laser-based ultrasonic testing (LUT). ET methods include eddy current testing (ECT), magnetic flux leakage (MFL) testing, and alternating current field measurement (ACFM). VT involves the use of two-dimensional (2D) or three-dimensional (3D) imaging techniques to obtain the surface condition of the rail.

Ultrasonic detection is mainly used to detect rail internal defects, but it has blind spots for surface and subsurface defects. Surface defects can be distinguished by reflection echoes of ultrasonic signals when they develop to a certain extent, but UT cannot evaluate the degree of defects or distinguish different types. ET and VT are the mainstream methods for detecting surface and subsurface defects. MFL and ECT use the permeability and conductivity effect of surface defects to detect them and can also evaluate the depth of defects.

However, the lift-off effect is a key issue that needs to be addressed in engineering applications of these technologies. VT is a relatively mature technique for detecting rail surface defects, but there are still challenges such as rail surface oil pollution and light interference that make it difficult to size small surface defects and evaluate depth. To improve defect identification, VT can be improved in four aspects: increasing sensor resolution, combining with inspection techniques such as UT and ET, conducting fusion analysis of 2D and 3D imaging, and continuing to innovate deep neural network techniques [8].

## 2. UT

**Figure 4:**
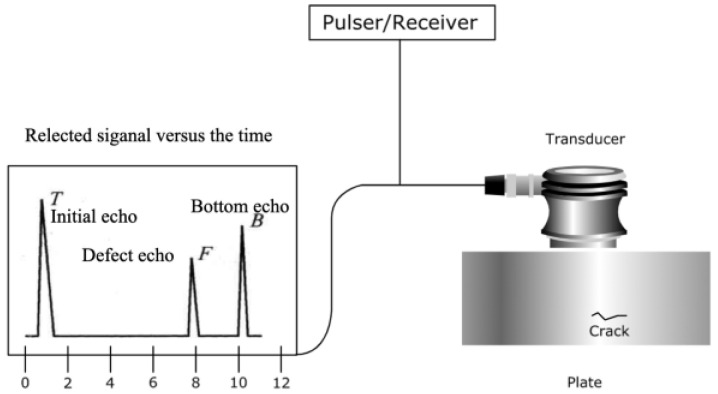
Basic principle of UT [11].

Nowadays, the ultrasonic testing technology of rail defects is developing and improving constantly and there are many mature applications in the field [9]. The UT system typically consists of an ultrasonic transducer, pulser/receiver, and display unit, as shown in Figure 4. The pulser/receiver is an electronic device that generates high-voltage electrical pulses to drive the transducer to produce high-frequency ultrasonic energy. The ultrasonic frequency for rail flaw detection is 0.2~25 MHz, and the most used is 0.5~10 MHz. The acoustic energy is then introduced into the material and propagates through the material as a wave. If there are defects along the wave path, some of the energy is reflected from the surface of the material back to the transducer. The reflected wave signal is converted into an electrical signal by the transducer and displayed on a screen for analysis [10,11]. 

### 2.1. Conventional UT

The conventional UT method for rail defect detection is based on an ultrasonic pulse-echo technique, which is mainly used for detecting rail internal defects. This technique is widely used in a variety of ultrasonic testing equipment, including portable walking sticks, dual-rail ultrasonic flaw detectors, and rail testing vehicles.

**Figure 5:**
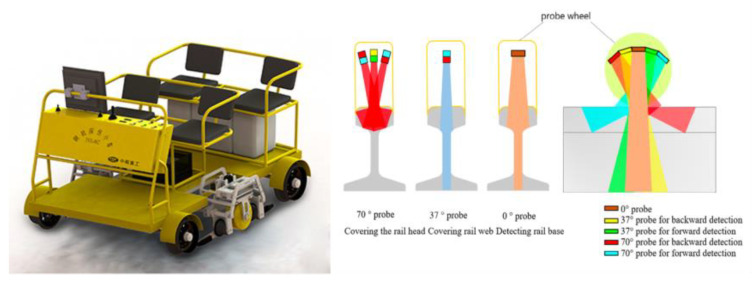
RUD-15 dual-rail flaw detector [12,13].

For example, as shown in Figure 5, the RUD-15 dual-rail flaw detector [12,13], developed by Shanghai Oriental Maritime Engineering Technology Co., Ltd., uses 18 pulse-echo probes arranged at fixed angles. Each probe corresponds to a specific rail flaw, as shown in Table 1. [14]. The information from the 18 channels is distinguished by the relative position and color of the B-scan image. This principle is also suitable for walking sticks and rail flaw detection vehicles.

**Figure 6:**
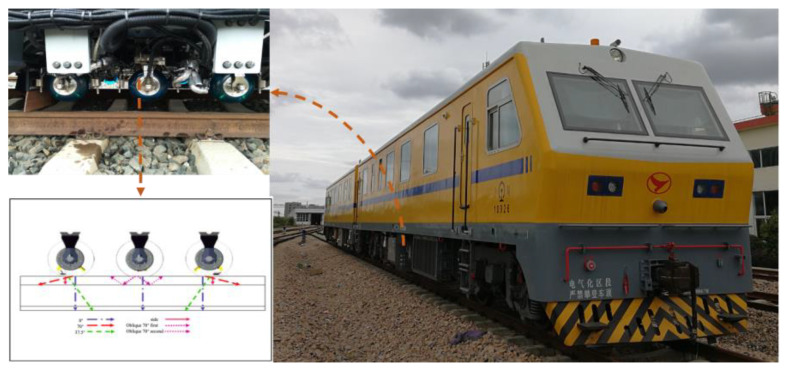
GTC-80X rail testing vehicle.

The GTC-80X rail flaw detection vehicle, developed by CHINA ACADEMY OF RAILWAY SCIENCES and equipped with UT probes, is shown in Figure 6. It is capable of detecting defects at speeds up to 80 km/h [15,16].

**Table 1:** Defects and corresponding detection probes [14,17].

Defect	Manual Defect		Dual-Rail Flaw Detector	Rail Testing Vehicle
	Type	Minimum Size	Testing Probe	
Rail transverse defect	Rail head flat bottom hole	*ϕ*4 mm	Oblique 70°	Oblique 70°
	Rail head cross hole	*ϕ*3 mm	Oblique 70°	Direct 70°
Rail fillet transverse crack	Rail fillet transverse grooving	R4H2	Oblique 70°	Direct 70°
Bolt hole crack	Bolt hole wire cutting	3 mm	0°, 37°	0°, 37°
Weld damage	Rail web cross hole	*ϕ*4 mm	——	37°
Bottom wave attenuation	Conical hole	*ϕ*10 × 10 mm × 120°	0°	——
Rail bottom transverse crack	Rail bottom transverse grooving	R4H2	37°	37°

The conventional UT is a widely used NDT method that offers several advantages for internal rail flaw detection. Firstly, it is a non-destructive and radiation-free technique. Secondly, it can be used for rail defect detection at high speed. Thirdly, the technique is well-established, and there are numerous successful applications across various industries [18]. However, the technique also has some limitations. For instance, the probes are fixed at specific angles, which restricts the detection of a broad range of rail defects. Additionally, it relies on coupling agents, which are susceptible to interference from extreme weather conditions and increased detection speeds. These shortcomings have prompted the development of more advanced ultrasonic methods.

### 2.2. PAUT

**Figure 7:**
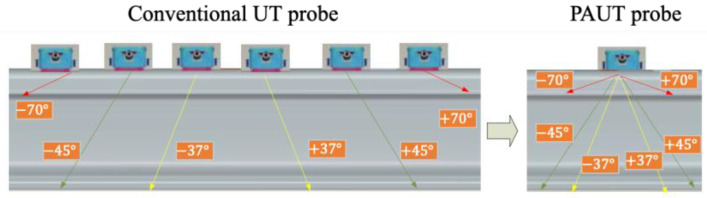
Scanning ability of conventional UT and PAUT probes [20].

Compared to conventional UT, PAUT utilizes a series of piezoelectric crystal transducers in a single assembly. Each transducer can be pulsed at different times for constructive interference and individually focused without the need to steer the probe across the component [19]. As shown in Figure 7, a fast-phased array probe can generate six angular beams at the same time, which is equivalent to six conventional probes simultaneously generating a fixed angle beam. Multiple angles allow simultaneous monitoring of the same area, ensuring a higher coverage [20]. Due to its powerful scanning capability, it can meet the requirements of full-section detection of 43, 50, 60, and 75 kg/m rails, which is extremely beneficial to rail defect detection in heavy-haul railways.

**Figure 8:**
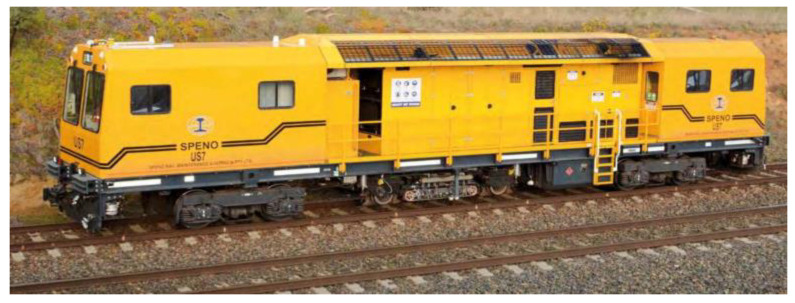
SPENO’s phased array rail detection vehicle [21].

SPENO has developed an ultrasonic rail testing vehicle, the US 7, which is equipped with a multi-element phased array unit, as shown in Figure 8. This advanced system replaces the traditional approach of using about ten probes to inspect the rail at a fixed angle. In addition, SPENO has designed a carrier trolley that protects the phased array unit from mechanical shocks. The US 7 is capable of reducing the time taken for sequential testing and can operate at speeds exceeding 80 km/h with a sampling frequency of 6 kHz, as depicted in Figure 8 [21]. The US 7 vehicle was assembled by SPENO in Perth in 2018 and transported to Sydney by road low loader.

**Figure 9:**
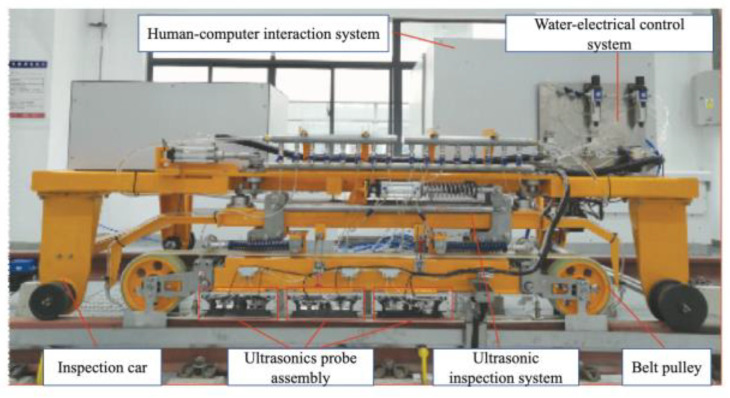
Fast phased array rail testing vehicle from Dazhun Railway Company [22].

Referring to the Zhuneng Group Dazhun Railway Company and Beijing Lead Time Science and Technology Co., Ltd.’s high-speed rail testing vehicle, shown in Figure 9, it is a novel type of rail inspection system based on fast phased array ultrasonic technology. Experimental results indicate that the multi-beam and variable angle ultrasonic layout of the PA70° probe meets the transverse defect detection requirements of various rail types. However, the UT70° probe, with its single wave beam and fixed angle layout, may miss detection due to rail type changes, wear, and mid-deflection. Therefore, the optimal layout scheme includes two steps: first, use the direct PA70° probe to replace the dip UT70° probe to adapt to different rail types and surface conditions. Second, the PA70° probe is excited to generate three different tilt angles simultaneously to address centering offset problems. The belt wheel layout is also used, which offers the advantages of both boot and wheel methods and ensures stable alignment during high-speed operation. The workshop experiments have performed well, with a defect detection rate of 100%, except for rail filter cracks at the 6 mm sampling interval, and meeting the 80 km/h detection speed requirement. This large rail flaw detector based on fast phased array technology can continuously detect over 350 km of rail lines and achieve a detection speed of 80 km/h or higher [22,23]. 

### 2.3. EMAT

**Figure 10:**
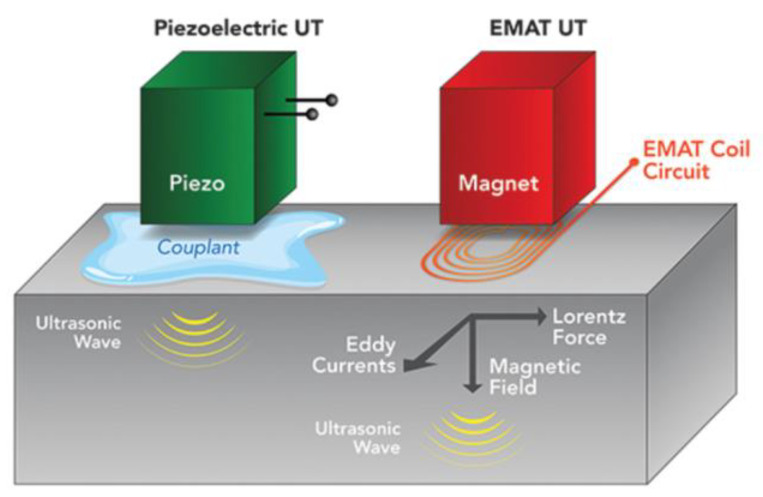
Comparison of piezoelectric UT and EMAT [24].

The principle of EMAT is the Lorentz force, magnetostriction, and magnetization force. Typically, EMAT based on the Lorentz force mechanism includes a coil of wire, a permanent magnet, and a conductive metal. There are two interacting magnetic fields generated by a coil of wire with high-frequency alternative currents and a permanent magnet relatively. Due to the high-frequency alternating current passing through the wire, the eddy current generated on the metal surface will be subjected to the Lorentz force in the two magnetic fields. As a result, the metal specimen will have periodic high-frequency vibrations, and particle vibrations will be superimposed to form ultrasonic waves. The comparison of the principle between the piezoelectric UT and EMAT is shown in Figure 10.

**Figure 11:**
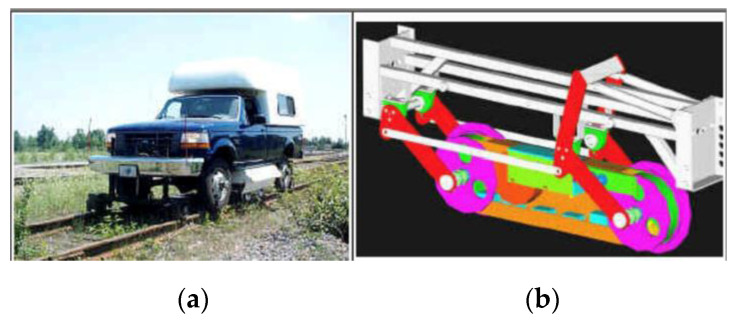
Rail test vehicle with the EMAT probe: (**a**) integrated mobile inspection system; (**b**) designed transducer carriage and holder [25].

Tektrend International Inc. from Canada designed a rail test vehicle with the EMAT probe carriage for real-time inspection. As shown in Figure 11, the carriage includes an enclosure, transducer assembly, and two levels of pneumatically activated articulations. Different EMAT configurations were tested to evaluate its performance and the detection capability of the system, detecting 12 types of defects and their locations and sizes [25].

**Figure 12:**
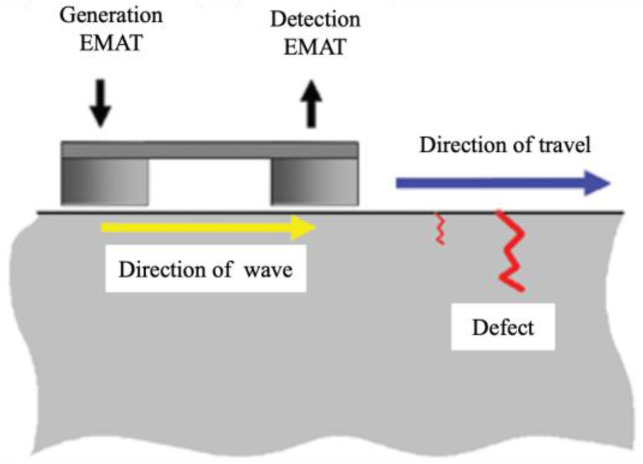
EMAT probe arrangement [26].

Edwards et al. [26] used a non-contact pitch–catch pulsed ultrasonic system to detect RCF cracks under high-speed condition. The EMAT layout of the system is shown in Figure 12, where one EMAT with a transducer that can move freely is used to generate ultrasonic waves that travel along the rail head. Then, a second EMAT detects these waves at a fixed distance of 150 mm. It can be concluded that EMAT is a feasible method for rail surface defect detection. 

**Figure 13:**
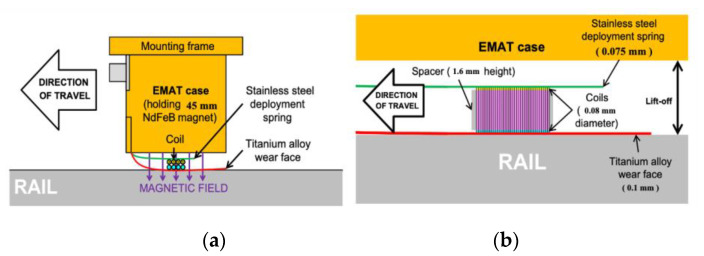
The side view of the design and EMAT coil design: (**a**) side view of the design; (**b**) EMAT coil design [27].

Compared to piezoelectric UT, EMAT is a completely non-contact technique and does not need any couplants. Moreover, it is less affected by surface conditions. However, lift-off can significantly affect the EMAT results, which limits its applications. Conventionally, EMAT is usually kept within a 3 mm lift-off in order to obtain a sufficient signal-to-noise ratio (SNR). Petcher et al. [27] designed a new EMAT that allowed the light and flexible EMAT coil to lightly touch the sample surface and hence skim it, while the heavier and bulkier magnet was allowed to vary over a larger range of lift-offs. A side-view of the design and the EMAT coil design are shown in Figure 13. The result shows that the proposed method can reach a lift-off of 10 mm. Zhu et al. [28] designed a rail flaw detection system consisting EMATs and their front-end circuits, a filed programmable gate array (FPGA) and its peripheral circuits, and a digital signal processor (DSP) and its peripheral circuits. The DSP was used to control the EMATs, and the FPGA implemented cumulative averaging and cross-correlation algorithms due to its flexibility and high processing speed. The results show that by using five channels of transducers, the system achieves comprehensive inspection of rails through four types of waves.

### 2.4. LUT

**Figure 14:**
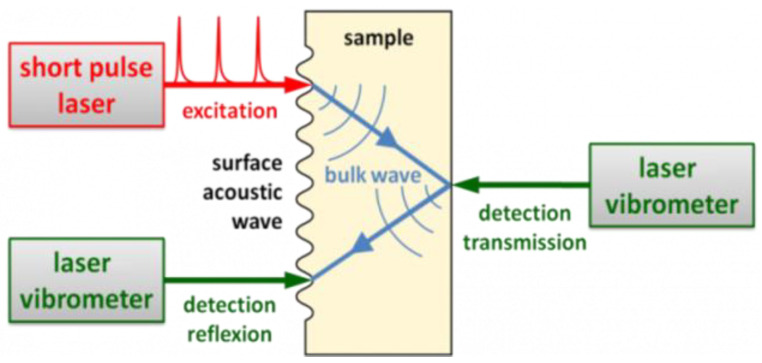
Principle of LUT [31].

LUT is a cutting-edge ultrasonic technique that utilizes a laser to generate and measure ultrasonic waves. It combines the sensitivity of ultrasonic inspection with the flexibility of an optical system. The principle of LUT is shown in Figure 14. Firstly, a pulsed laser generates various types of ultrasonic waves through a thermoelastic process or ablation. These waves are diffused into the material and reflected by a laser vibrometer against structures and defects. The measured signals are then processed to produce and display the required information [29,30]. 

LUT stands out as an excellent approach for its various advantages. Firstly, like the EMAT, LUT is also a completely non-contact NDT method and needs no couplant, which reduces the requirements for rail surface roughness and cleanliness. Secondly, its transduction is performed by light, and it is suitable for the detection of remote distances and extreme environmental conditions. Thirdly, the pulsed laser can generate ultrasonic waves of various modes, so it can not only be used to detect the internal defects of rail, but also provide the possibility for rail surface defect detection. However, laser ultrasound technology still has some limitations such as low efficiency of photoacoustic energy conversion, weak ultrasonic signal, and high cost of detection equipment [29,32].

**Figure 15:**
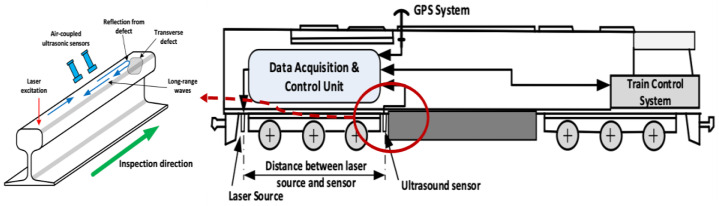
Moving vehicle design with LUT system [33].

As shown in Figure 15, the Centre for Railway Engineering of Central Queensland University proposed a conceptual design with a laser-induced ultrasonic guided wave detection technology that can be deployed on a moving vehicle. A pulsed laser source is used to generate ultrasonic guided waves and air-coupled ultrasonic sensors are cooperated to sense the waves. Before the field application, finite element simulations and preliminary tests are carried out and it can be concluded that a 2 MHz ultrasound signal can travel over a 2.4 m distance along the rail head and be reflected. Moreover, the related work establishes the strength of the technology for rail foot flaw detection [33,34].

**Figure 16:**
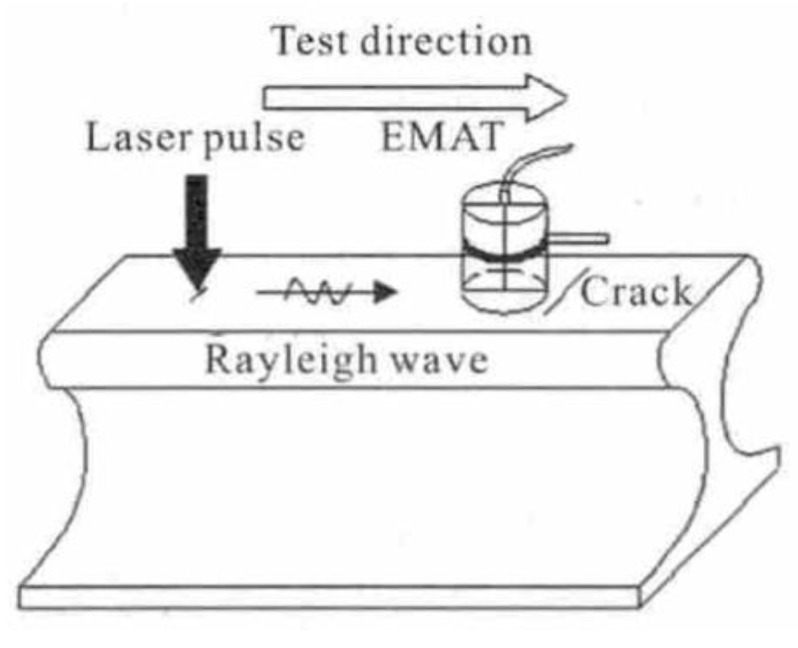
Overall architecture of the experiment proposed by Nan et al. [35].

Nan et al. [35] investigated a novel LUT-based detection system for the detection of rail surface cracks of not less than 0.5 mm, as shown in Figure 16. The optimal value of lift-off is about 1–3 mm. It utilizes high-energy laser pulses to generate Rayleigh waves, which can be reflected and converted into shear waves when they pass through regions with different acoustic impedances. The EMAT probe is then used to receive the signal from the waves. To achieve a better SNR, the EMAT probe coil is designed as a four-layer Printed Circuit Board (PCB) with a butterfly structure. Tests indicate that the lift-off distance between the EMAT probe and the rail steel specimen should be less than 3 mm.

**Figure 17:**
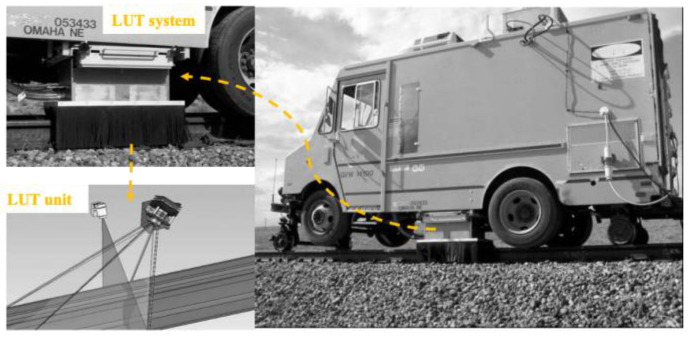
Hi-rail vehicle with the laser-based system [36].

A laser-based ultrasonic inspection system is developed by Tecnogamma SPA and evaluated by the Transportation Technology Center, Inc. form Colorado of North America (TTCI). It utilizes a high-energy laser to introduce ultrasonic signals and air-coupled transducers to monitor various wave modes. The rail flaw inspection prototype is a hi-rail vehicle installed system, as shown in Figure 17. It can detect the following types of defects: head transverse defect, horizontal split head, vertical split head, shelling, horizontal split web, piped web, and base transverse defect at speeds of 32 km/h or higher [36]. 

### 2.5. Summary of UT Methods

UT methods are commonly used for rail internal defect detection. Conventional piezoelectric transducer UT is a well-established technique and is widely used in field applications. However, it has various limitations for full rail coverage detection and detection of different types of rails. Benefiting from the multi-angle scanning capability of the probe, PAUT is gradually attracting more attention in the testing field, and has some field applications on heavy-haul railways. EMAT and LUT are both at the cutting edge of UT technique, and they are both non-contact and possess the ability to detect rail surface defects. However, limitations such as lift-off effects and photoacoustic energy conversion efficiency need to be addressed before they can be applied in the field.

## 3. ET

### 3.1. ECT

**Figure 18:**
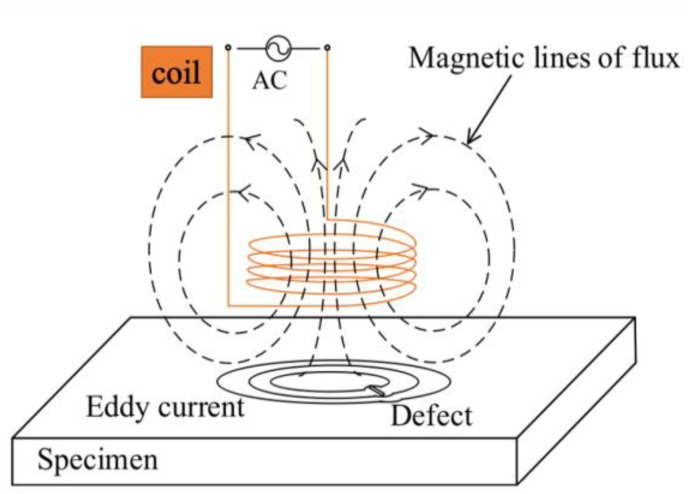
Principle of ECT [38].

ECT uses the principle of electromagnetism to detect flaw in conductive materials. The principle of conventional ECT is shown in Figure 18. Alternating current (AC) flows through the wire coil and generates an oscillating magnetic field, called the primary magnetic field. According to the principle of electromagnetic induction, eddy currents will be induced into the specimen, and they generate their own magnetic field, called the secondary magnetic field. This secondary magnetic field opposes the primary magnetic field, and we can measure this effect using the impedance between the coil and the specimen. If there are defects, the distribution of eddy currents will change and cause a change in impedance [37,38]. In general, the penetration depth δ is as the following equation. It refers to the value when the magnetic field intensity and eddy current density are reduced to 1/e of the surface. δ is one of the decisive factors in the detection depth of subsurface defects. δ is the penetration depth. f is the frequency. μ is the permeability. σ is the electrical conductivity [39,40].

$$\delta =\sqrt{\frac{1}{\pi f\mu \sigma }}$$

Currently, ECT is one of the mainstream methods for rail surface and subsurface defect detection. Many portable devices based on the ECT principle are applied in the field. 

**Figure 19:**
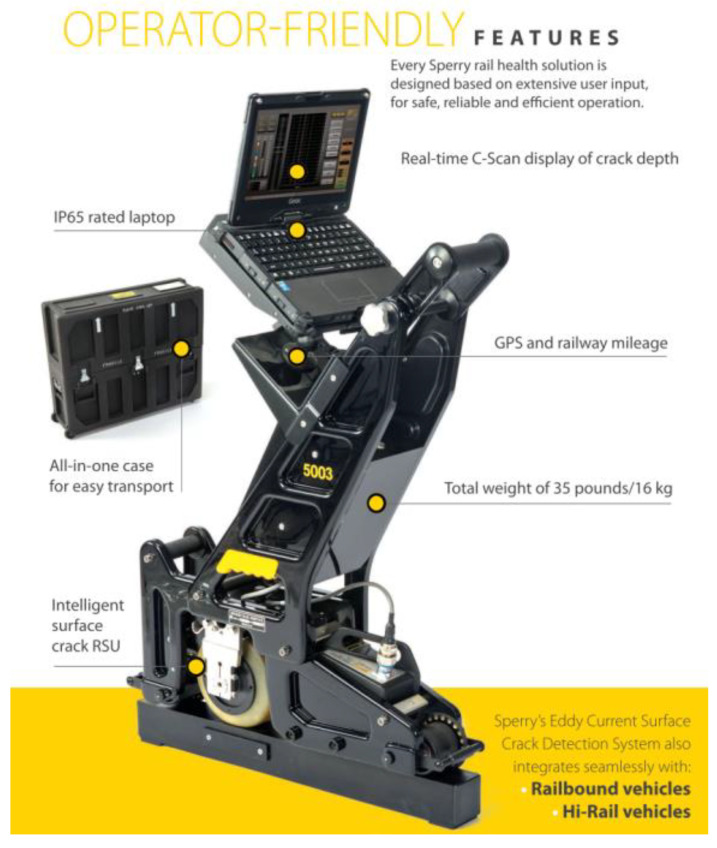
Sperry’s eddy current walking stick [41].

Figure 19 shows the eddy current walking stick of SPERRY, which is equipped with roller search units (RSUs) with built-in digital processing to maintain constant contact with the rail. It provides full coverage of the entire rail head for the detection of RCF cracks and other surface damage to the rail. There are some special features shown below [41].

**Figure 20:**
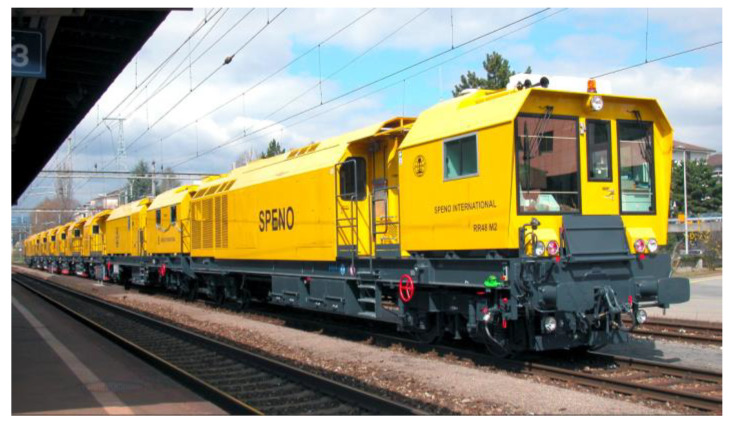
Grinding Train of SPENO International SA [42].

Meanwhile, some larger pieces of equipment have been developed by the eddy current principle. Figure 20 shows the grinding train of SPENO International SA with an eddy current test system. The system was developed and optimized at the BAM together with partners, especially for head check detection [42,43].

**Figure 21:**
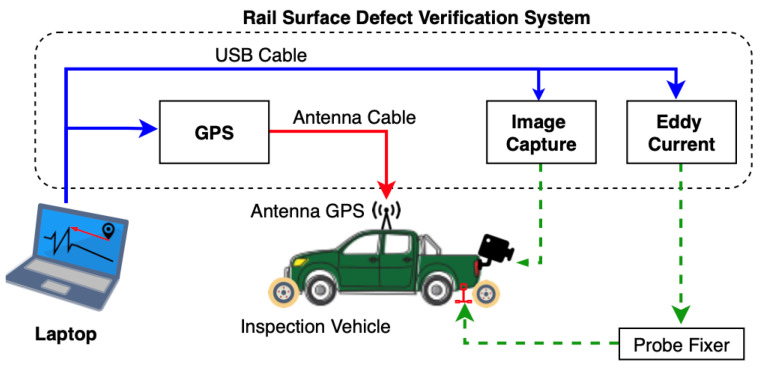
RSDVS diagram proposed by Alvarenga et al. [44].

Alvarenga et al. [44] proposed the Rail Surface Defect Verification System (RSDVS) and integrated it into the inspection vehicle based on eddy current. The system diagram is shown in Figure 21. There are two eddy current probes installed on the vehicle and they are at 2 mm from the rail surface. Generally, the vehicle travels under analysis with a constant speed of 5~30 km/h, but to decrease noise due to trepidation, the recommendation is at a lower speed.

ECT is a non-contact NDT method that is capable of detecting surface and subsurface cracks as small as 0.5 mm [37]. However, there are some limitations to its widespread use in high-speed conditions. There are two main reasons for this: from a principal point of view, the lift-off, which is the distance between the probe and the rail head, has a significant impact on the detection results. High speeds can lead to variations in lift-off, potentially inconsistent results, and relatively low confidence levels. From the perspective of signal analysis, it is difficult to find an accurate correspondence between the signal and the defect, and to use the information from the signals to evaluate the defect. In recent years, there have been some relevant studies on eddy current signal processing and analysis [8,45,46].

On the basis of the traditional ECT principle, eddy current array (ECA) [47], pulsed eddy current (PEC) [48], and eddy current pulsed thermography [49] and other derived ECT technologies have gradually become a hot topic in the research field, providing novel solutions for rail surface and subsurface defect detection.

### 3.2. MFL

**Figure 22:**
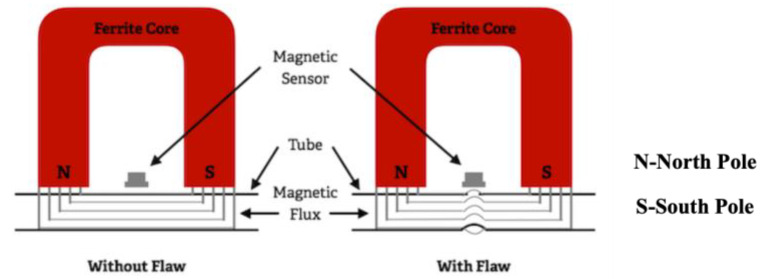
Principle of MFL [51].

MFL uses electromagnetism to inspect for flaws or material degradation in ferromagnetic materials. The principle of MFL is shown in Figure 22. A magnetizer and magnetic sensor form the MFL probe. MFL magnetizes the specimen using a magnetizer, which can be a magnet with a ferromagnetic yoke or a magnetizing excitation coil. If there is a flaw, the permeability of the magnetized specimen will change, and the induced magnetic field will be distorted. Then, the formed leakage magnetic field is measured with a magnetic sensor to obtain information about the defect [38,50]. 

**Figure 23:**
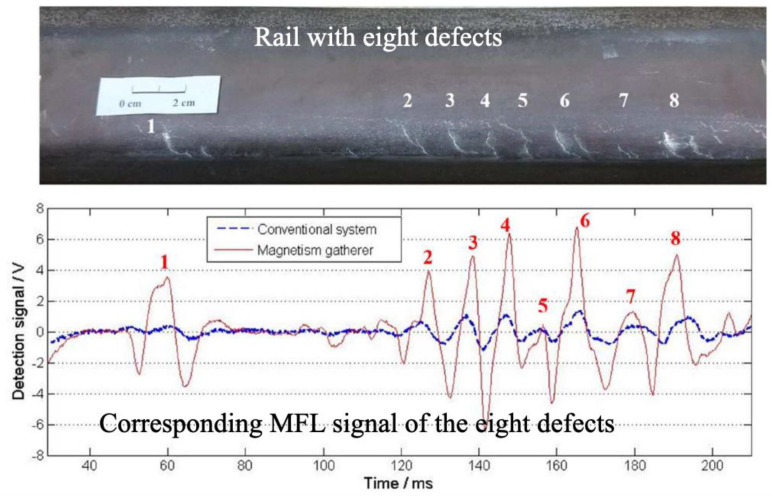
Comparison of test results of an FMG system and a conventional system [54].

The lift-off is the vertical distance between the probe and the detected rail surface. MFL signals are significantly influenced by the lift-off, rail surface state, and many other factors. The lift-off is significantly affected by the vibration during the operation. Therefore, in some cases, the lift-off effect limits the high-speed detection application of MFL [52]. Meanwhile, as the magnetic field changes with time and position, there will arise eddy currents. Furthermore, they prevent the magnetic field from penetrating inside the rail, which is also a reason for limited high-speed rail inspection using MFL. Therefore, it is proposed to increase the pole distance within the magnetizing system to increase the time of the exposure of the rail area to the magnetic field [53]. Jia et al. [54] presented an MFL method based on a ferrite magnetism gatherer (FMG) to enhance the MFL signals and it is suitable for the detection of small defects on the rail surface. Figure 23 shows an example of the detection comparison of the proposed FMG system and the conventional system. 

**Figure 24:**
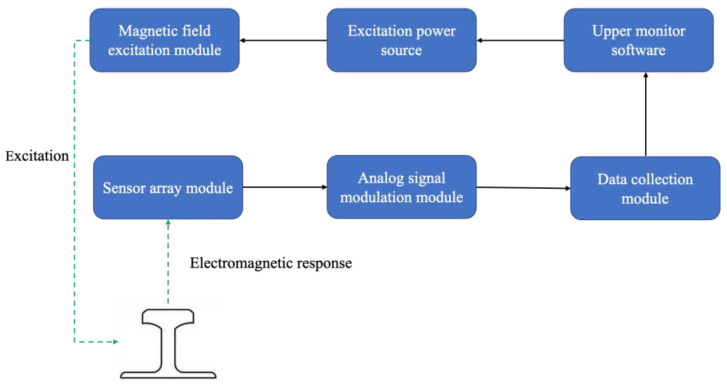
Overall MFL testing system [55].

Xu et al. [55] designed an MFL testing system that is mounted on the GTC-80X rail flaw detection vehicle, as shown in Figure 24. In laboratory conditions, it can detect the top surface damage with a width of 0.2 mm at a high speed of 50 m/s. In the field test condition, the maximum speed is 40 km/h. Through the testing test of the artificial opening of the calibration line, it is shown that the vehicle-mounted magnetic leakage detection system can accurately detect the artificial defects on the calibration line and distinguish the size of defects of different sizes.

### 3.3. ACFM

**Figure 25:**
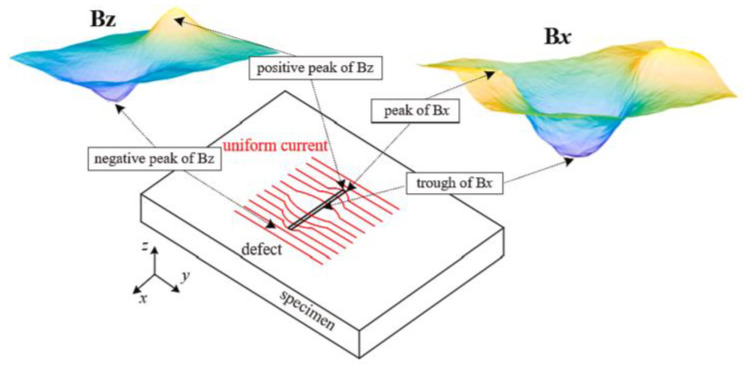
Principle of ACFM [38].

ACFM is an electromagnetic technique used for the detection and sizing of surface-breaking cracks in metallic components and welds. It combines the advantages of the alternating current potential drop (ACPD) technique and EC testing in terms of defect sizing without calibration and the ability to work without electrical contact, respectively. The principle is shown in Figure 25, where there is a uniform alternating current on the specimen, and the magnetic field in the x and z dimensions have abnormal characteristics if there are defects. According to the distribution of the peaks of Bx and Bz signals, the location and size will be detected [38].

**Figure 26:**
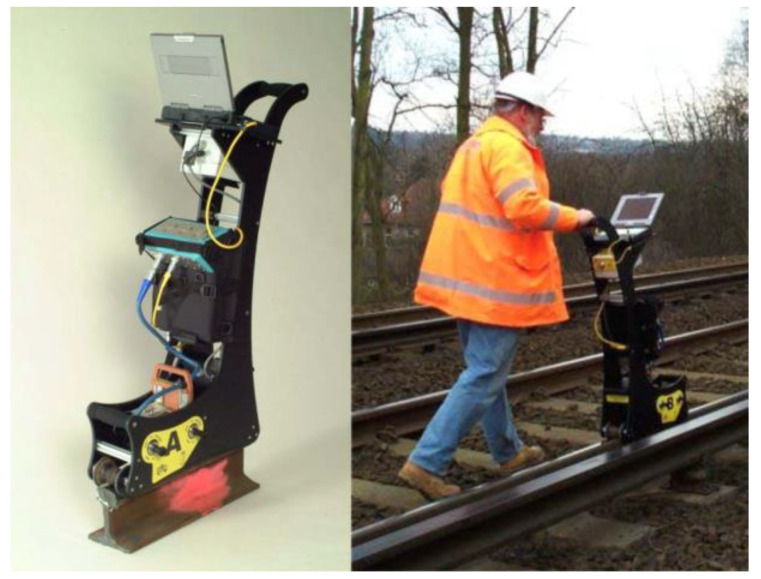
ACFM rail walking stick [56].

Figure 26 shows a pedestrian ACFM walking stick which is certified for use on the UK rail network. It can work at speeds of 2–3 km/h for at least 8 h from its own power source. It was proposed that it was necessary to consider the mathematical modeling and its relevance to the gauge corner cracking (GCC) crack form for sizing and empirical correction factors would be applied for the crack type [56]. Nicholson et al. [57] used a FEM model developed with COMSOL Multiphysics to identify the relationship between ACFM signals and RCF defects. By using a sizing algorithm based on a semi-elliptical, the defect would be undersized by 34.4%.

**Figure 27:**
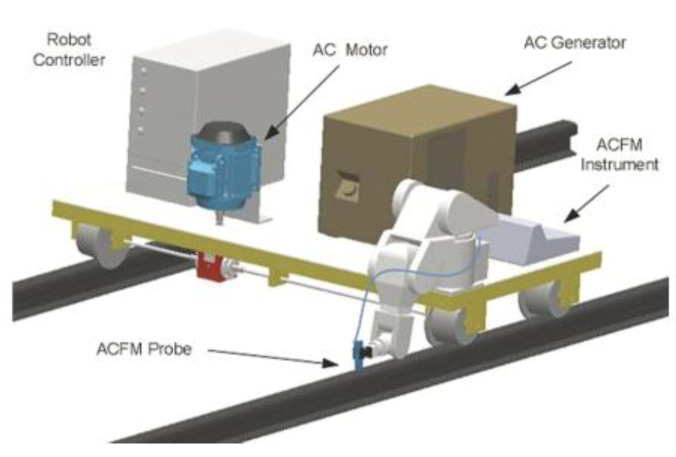
ACFM autonomous rail inspection vehicle proposed by Rowshandel [59].

Papaelias et al. [58] carried out high-speed ACFM inspection tests using a micro-pencil probe. It was found that under a constant lift-off of 0.8 mm, the signal remained largely unaffected by the increases in speed. However, the ACFM signal for a given defect showed a square reduction as the lift-off increased (2, 3, 4, and 5 mm) at the same speed range. Rowshandel et al. [59] developed a robotic system for RCF defect detection based on ACFM. The autonomous inspection vehicle is shown in Figure 27. It can achieve inspection speeds up to 10 km/h. The research shows that, if the lift-off exceeds 4 mm, the test results will be inaccurate.

### 3.4. Summary of ET Methods

In summary, ECT, MFL, and ACFM are non-contact NDT methods based on electromagnetic theory, and they are suitable for the detection of rail surface and subsurface defects. It is clear from the research and product review that they are all affected by lift-offs, which needs to be addressed for high-speed detection applications. They have their own characteristics and limitations. Firstly, they are suitable for different detection speeds. The ECT signal decreases with an increase in speed, and therefore it is more suitable for low- and medium-speed detection. The MFL signal increases with an increase in speed, and therefore it is more suitable for high-speed detection. The ACFM signal remains essentially constant with an increase in speed, and therefore it is more suitable for the evaluation of defect parameters [38]. Secondly, ECT and ACFM are susceptible to skin effect. Therefore, MFL has the advantage of detecting deeper defects compared to ECT and ACFM.

## 4. VT

VT is a widely used NDT method across all industrial fields. It is usually used to detect flaws that are visible to the naked eye. Therefore, compared with ET methods, VT is more suitable for detecting surface defects with relatively large area and shadow depth. VT is usually classified as 2D VT and 3D VT. Generally, 2D VT refers to the use of linear array cameras to obtain images of the rail, and then perform data processing to identify defects. In contrast, 3D VT refers to the use of a three-dimensional laser radar or camera to scan the rail from different angles to obtain the condition of the rail.

**Figure 28:**
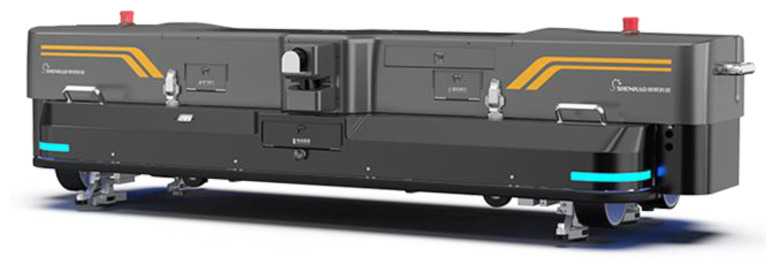
RIIS005 Railway Inspection Robot of Hangzhou Shenhao Technology Co., Ltd. [60].

Many forms of VT devices are used for rail surface defect detection. They can be roughly divided into three categories: railway inspection robots, UAVs, and vehicle-mounted devices. For example, Figure 28 shows the RIIS005 railway inspection robot developed by Hangzhou Shenhao Technology Co., Ltd. form Hangzhou of China [60]. It utilizes CCD cameras to detect rail surface defects such as burn and shelling. The robot weighs 118 kg, the inspection speed can reach 5 km/h, the expansion speed can reach 15 km/h, and the battery cruise time is greater than 4 h, which is suitable for daily inspection.

**Figure 29:**
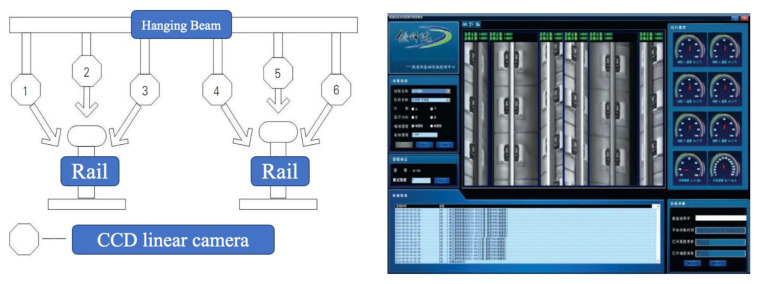
Onboard track inspection system [62].

The widely used VT devices are vehicle-mounted, and it is an efficient way to operate high-speed detection for the in-service rail. Figure 29 shows an onboard track inspection system based on computer vision proposed by the China Academy of Railway Sciences (CARS) [61]. The rail surface image data are acquired by six linear cameras from different angles at an equal interval of 1.6 mm and analyzed with ML algorithm. The detection rate of rail surface burns is above 95% and the speed can reach 160 km/h. GTC-80 rail flaw detection vehicles began to be equipped with the onboard track inspection system after 2013 [62].

**Figure 30:**
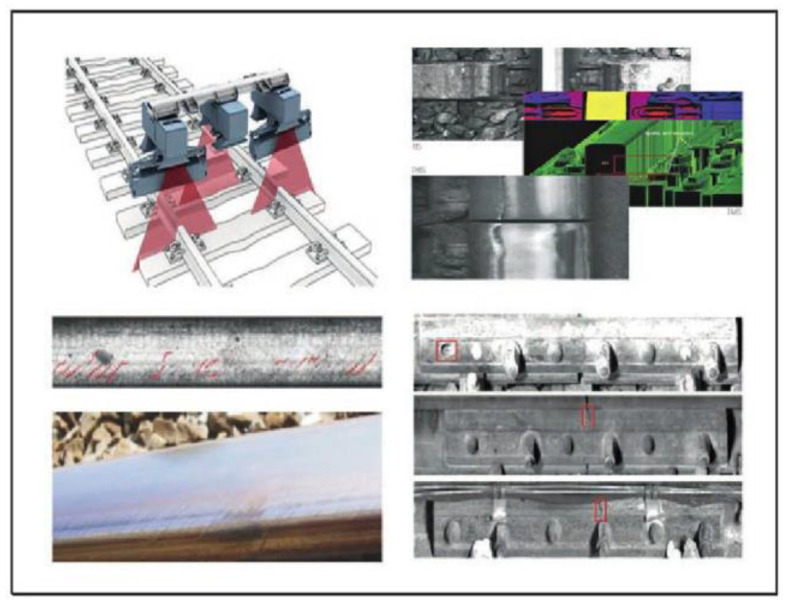
Structure and data effect of the track inspection system of Mermec in Italy [62].

Figure 30 shows the track inspection system developed by Mermec, Italy. It adopts non-contact detection technology based on optics and adopts a linear array camera to collect line pictures. The car is also equipped with high-power air-cooling equipment to dissipate heat for the detection equipment under the car. The system detection speed reaches 160 km/h [63]. The system was introduced by the Qinghai-Tibet Railway Company in 2006 and put into use in 2009 [62].

**Figure 31:**
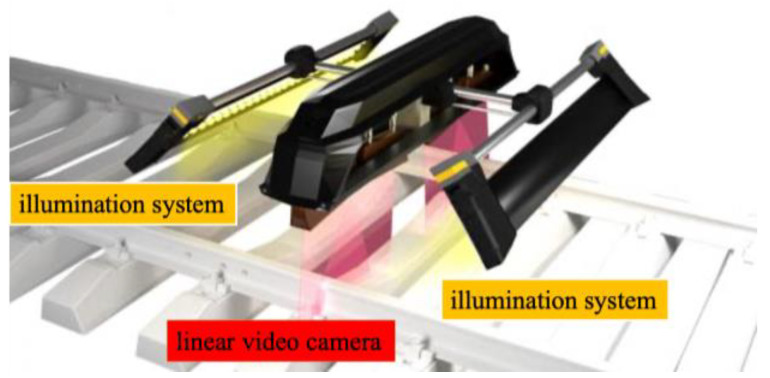
TVEMA VT system [64].

TVEMA produced a VT system using a linear video camera, as shown in Figure 31. It is used for the detection of rail surface flaws such as rail shelling and damage to welded joints. Its compact design makes it possible to install equipment on the car bogie of any rolling unit. The linear cameras can shoot at a high resolution of 1 mm/px at speeds up to 400 km/h [64].

Generally, VT is mainly used for large-size defect detection. In most cases, it appears in the form of a modular system design that can be installed on different forms of equipment. It is usually used in cooperation with other methods to accomplish two or more detection tasks at a time. In recent years, VT for rail defects is gradually focusing on 3D technology. For example, there are several studies on combined linear laser and camera for rail profile inspection [65,66]. The detection performance of both 2D and 3D VTs relies heavily on the method of processing the acquired rail surface data. Artificial Intelligence (AI), Machine Learning (ML), and Deep Learning (DL) are gradually being combined into VT, which then forms the subfield of machine vision. There are several related studies that apply machine vision to improve detection accuracy and speed [67,68,69].

## 5. Integrated Methods

As we can see, each NDT method has its drawbacks and limitations. Therefore, it is essential to combine two or more NDT methods for comprehensive detection in various industries [70,71,72,73]. By combining various methods, a synergetic effect will be created to overcome the limited detection based on the results of only one testing method.

**Figure 32:**
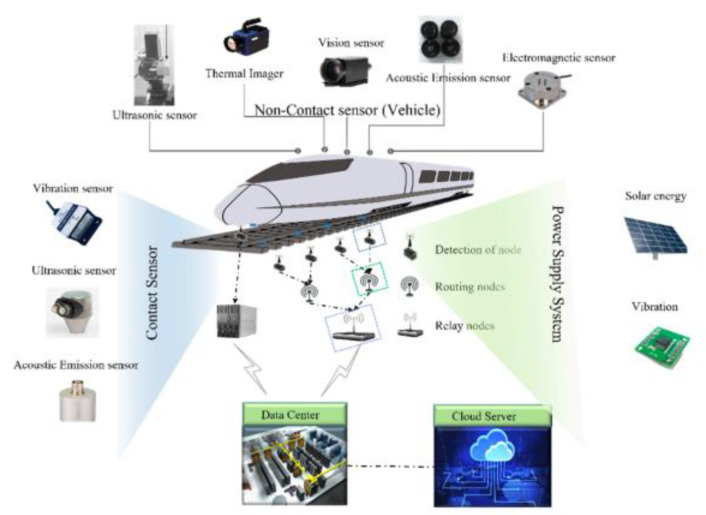
An integrated rail defect detection system [74].

With the development of high-speed and heavy-haul railways, there are more stringent requirements for detecting the condition of rail infrastructure, including rail defects. It is of vital importance for operating comprehensive, reliable, and cons-effective rail defect detection by using integrated methods and systems, as shown in Figure 32 [74].

**Figure 33:**
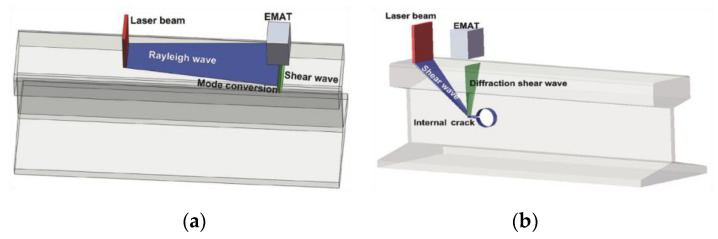
Diagram of internal and surface defect inspected by the hybrid laser–EMAT method: (**a**) diagram of testing surface defect based on the method of wave mode conversion; (**b**) diagram of internal defect inspected by the method of shear wave diffration [75].

The Centre of NDT Engineering and Technique in Shandong uses a novel method that combines laser generation with EMAT detection to achieve the detection of rail surface, subsurface, and internal defects. As Figure 33 shows, a Rayleigh wave is applied to inspect the surface defects, while a shear wave is selected to detect the internal defects in the rail. The wavelet soft-threshold method (WSTM) is introduced to reduce noise and improve the testing accuracy and SNR. The detection results of surface cracks, surface vertical holes, bolt hole cracks, and web holes verified the effectiveness of the proposed method. The relative errors of inspection for surface defects and internal defects were all less than 10%. The described hybrid laser–EMAT system performs well and provides an effective solution for rail defect detection [75].

**Figure 34:**
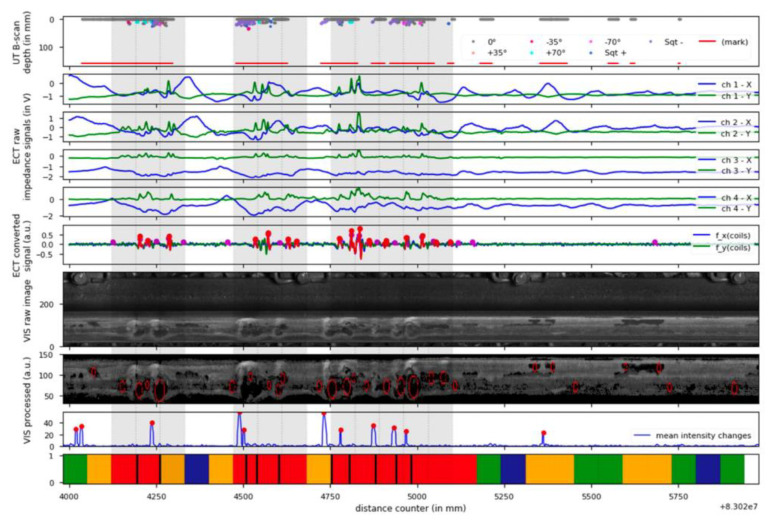
Example of raw data from the integrated system based on [77].

Eurailscout Inspection and Analysis [76,77] optimized the RCF identification and classification by the usage of ultrasonic B-scans, eddy current signals, and the rail head images, which were recorded by systems combined with UT and ECT probes, and surface imaging by video (VIS). The UT system is equipped with special ‘squat probes’ designed by Sonimex b.v. The ECT system is developed by BAM and PLR and includes an eight-channel recording device, a signal processing unit, and eight HC10 eddy current probes. The VT system is a rail inspection video system from bvSys. The integrated system is already installed on UST 02 inspection train and Figure 34 shows an example of raw data from the system. The results show that, for squat detection, the UT is best among the individual methods. It is more suitable for squat detection by combining UT and VT instead of all three methods.

**Figure 35:**
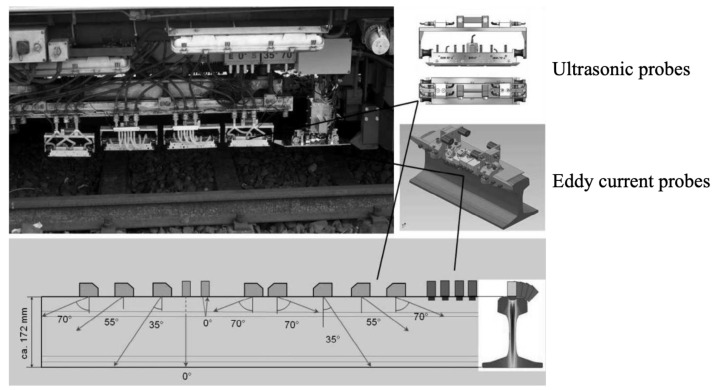
Probe arrangement of ultrasonic and eddy current integrated system [78].

An integrated system with ultrasound probes and eddy current sensors was implemented into the SPZ1 inspection train by PLR, and it is operated by Deutsche Bahn AG. The probe arrangement is shown in Figure 35. The ten ultrasonic probes are GE type in standard housing and keep the 0.2 mm gap between the rail head and probes. The four eddy current sensors are HC-10 type and are situated at a distance of 1 mm to the UIC60 rail surface. It uses the unique Glassy-Rail-Diagram to represent the conventional B-Scans. This implies automated data analysis tools based on trained neuronal networks and fuzzy logic. It can reach very high speeds above 80 km/h [78]. 

**Figure 36:**
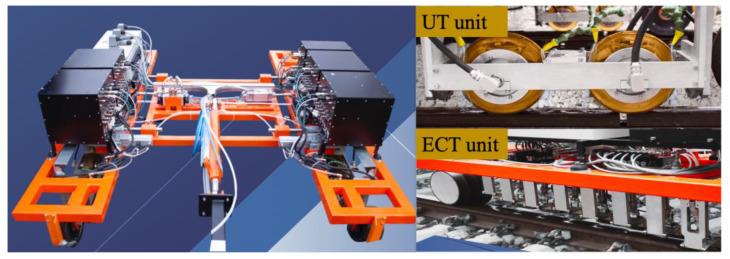
Rail flaw detection trolley based on UT and ECT [79].

OKOndt GROUP [79] developed the OKOSCAN 73HS system for automated high-speed testing of rails. The system is designed for UT and ECT of railway tracks at a speed up to 40 km/h and detection of all the defects specified in the UIC 712 R (International Union of Railways Code of Rail Defects, from UIC, Paris, France). The flaw detection trolley equipped in the system is shown in Figure 36. The ultrasonic rail test unit incorporates four wheel probes, and each probe includes six transducers. The eddy current rail test unit is equipped with sixteen eddy current probes (ECP), eight for each rail.

GTC-80IIX rail flaw detection vehicle is developed by equipping the UT and ET system based on the first generation of self-developed rail flaw detection vehicle. At the same time, it is equipped with an imaging system for rail surface and rail profile detection, which can realize comprehensive detection. This type of vehicle has been used in two railway bureau group companies in China. The properties of the different combinations of rail defect inspection methods are concluded in Table 2.

As we can see, the current integrated methods are mainly used to detect the rail surface and subsurface defects and exit different limitations of their own. It is necessary to design a robust and effective system for comprehensive rail detection.

## 6. Trend and Prospect of Rail Detection

The trends of rail detection mainly focus on the following two aspects. Firstly, the detection speed is gradually increasing. With the development of high-speed and heavy axle loads, the requirements of detection frequency and accuracy are higher. The skylight time is limited. Therefore, the efficiency needs to be improved and the detection should be operated at a higher speed.

Secondly, various NDT methods will be integrated to achieve more comprehensive rail detection. As Table 3 shows, whether UT, ET, or VT, they are just suitable for specific types of rail defects. It is impossible to make a comprehensive overall evaluation of the rail state if it only utilizes one method.

**Figure 37:**
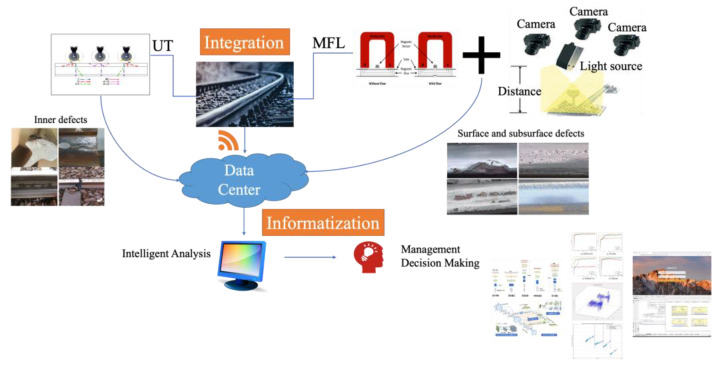
A novel integrated rail defect detection method.

In order to meet the requirement of high-speed and comprehensive detection, this paper proposes a detection method that integrates various sensors and provides the following data analysis to guide the maintenance plan. UT is the best choice for internal defect detection. Among the various UT methods, the technology of conventional UT is mature and experience in field application is rich. Therefore, the conventional UT is selected to be one of the methods in the proposed design. For surface and subsurface defect detection, MFL is more suitable for high-speed operation and has no skin effect compared with ECT. To make the detection more refined, VT is also integrated, it is popularized in various industries, and its relative image processing methods are mature. It could make surface defect detection more credible. Therefore, MFL and VT are chosen to detect surface and subsurface defects. As shown in Figure 37, UT, MFL, and VT are the main methods that guarantee comprehensive rail detection. After the integrated detection, it is essential to cooperate with the following intelligent data analysis and management. The detection data from different sensors need to be gathered into a data center and continue the analysis. For intelligent data analysis, it is advised to use the deep learning method to classify the images and signals and then identify the types of defects. Meanwhile, it is also feasible to utilize historical data to find the rules of the defect changes and then predict the state of the rails. Finally, the aim of the detection is to guide the maintenance plan. A management system and platform should be developed and established for the presentation of the analysis results and for providing guidance and recommendations for workers. 

## 7. Conclusions

Rail defects include internal defects, surface defects, and subsurface defects. The harmful degrees of the diverse rail defects are different, as well as methods/technologies to maintain/repair them. The rapid search, identification, and evaluation of rail inspection are of great significance for healthy rail maintenance.

Conclusions. Based on the need for rapid search, identification, and evaluation of rail defects, this paper summarized the advantages and disadvantages of NDT methods, such as UT, ET, and VT, as well as their applications to rail inspection in the field track.

- As the main technical means for detecting internal defects in rails, traditional UT technology has the advantage of the mature application, simple operation procedure, and low cost, although the disadvantages are that the results are easily affected by coupling water and vibration during quick detection. This means that UT technology will still be the main means for internal rail defect detection in the future.
- For ET technology, MFL is more suitable for detection at high speed. Due to being not affected by eddy current effects, the detection depth is deeper than ECT and ACFM testing technology. In the future, it will become a major technical means to compensate for the blind spots in ultrasonic testing.
- Visual inspection technology has the advantages of intuitive, non-contact, and mature applications, which will play an important role in the detection of rail surface defects.

Perspectives. Based on reviewing the advantages and disadvantages of the above detection techniques, it is essential to integrate different NDT methods to achieve comprehensive and cost-effective detection. This paper advises utilizing UT, MFL, and VT for comprehensive detection. MFL and VT technology are used to comprehensively detect and evaluate surface and subsurface defects, which will prevent shallow surface defects from developing into transverse cracks. Real-time maintenance will be beneficial to extending the service life of the rail. Particularly, an ultrasonic flaw detection system is used to detect internal defects in the rail, which will prevent rail breakage and ensure transportation safety.

## Figures and Tables

**Table 2:** Properties of the different combinations of rail defect inspection methods.

Method	Application	Test Type	Inspection Speed	Characteristic
LUT + EMAT [75]	Internal and surface, subsurface defects	Laboratory	No mention	The wavelet soft-threshold method (WSTM) is introduced to reduce noise and guarantee a good SNR.
UT + ECT + VT [76,77]	RCF	Field	70 km/h	The benefit of integrated methods for squat classification is evident and the combination of two methods performs better than the three methods.
UT + ECT [78]	Internal and surface, subsurface defects	Field	80 km/h	A unique Glassy-Rail-Diagram is used and cooperation with automated analysis.
UT + ECT [79]	All the defects specified in the UIC 712R	Field	40 km/h	Pneumatic equipment mounted on the trolley is used for automatic adjustment to the track width. It is allowable to position each transducer on its own tested area.

**Table 3:** NDT methods for rail defect detection.

NDT Method		Defects	Characteristics and Limitations	Effectiveness
UT	Conventional UT	Internal defects	The technique is well-established and field experience is rich. The probes are fixed, and the detection range is restricted. It relies on coupling agents and the surface condition and speed will affect the results.	The detection rate of 100% as the standards require at a high speed of 80 km/h [15,16].
	PAUT		Multiple angles ensure a higher coverage rate and suitable for different types of rail. It needs coupling agents, and the field application is low.	The detection rate of 100% as the standards require at a high speed of 80 km/h, except for rail filter cracks at the 6 mm sampling interval [22,23].
	EMAT		It does not need any coupling agents and is a completely non-contact technique. It is affected by the lift-off effect. The technology is not mature enough and relative application is low.	The lift-off could reach 10 mm for sufficient SNR [27].
	LUT		It does not need any coupling agents and is a completely non-contact technique. It has low efficiency of photoacoustic energy conversion, weak ultrasonic signal, and high cost of detection equipment. The technology is not mature enough and relative application is low.	Surface cracks with width > 0.5 mm at best lift-off 1~3 mm [35].
ET	ECT	Surface and subsurface defects	It is a non-contact NDT method. It can detect cracks as small as 0.5 mm. It is susceptible to skin effect. ECT signal decreases with an increase in speed, which restricts the application at high speed.	Surface cracks width > 0.5 mm at a speed of 5~30 km/h [44].
	MFL		It is a non-contact NDT method. It also has the lift-off effect. It can detect deeper subsurface defects compared with ECT. It is more suitable for detection at high speed.	Surface defects width > 0.2 mm at a high speed of 50 m/s [55].
	ACFM		It is a non-contact NDT method. It is susceptible to skin effect. The technology is not mature enough and relative application is low.	Spark-eroded notches 2 and 4 mm deep at speeds up to 121.5 km/h [58].
VT		Surface defects	It is a non-contact NDT method. It is only suitable for detecting surface defect with large size. The technique is mature enough and there are many references from many other industries.	Surface defects with large size or shelling at speeds up to 400 km/h [64].
Integrated Methods	LUT + EMAT	Internal and surface, subsurface defects	The integrated methods are mainly focused on surface and subsurface defect detection. Its aim is to make the results more reliable. There are few applications about the detection for both internal and surface and subsurface defects at high speed. MFL is not considered in the integration.	—
	UT + ECT + VT			
	UT + ECT			

## Data Availability

The data presented in this review can be requested from the corresponding author or the first author.
